# Butyric acid, a gut bacteria metabolite, lowers arterial blood pressure via colon-vagus nerve signaling and GPR41/43 receptors

**DOI:** 10.1007/s00424-019-02322-y

**Published:** 2019-11-15

**Authors:** Maksymilian Onyszkiewicz, Marta Gawrys-Kopczynska, Piotr Konopelski, Marta Aleksandrowicz, Aneta Sawicka, Ewa Koźniewska, Emilia Samborowska, Marcin Ufnal

**Affiliations:** 1 Department of Experimental Physiology and Pathophysiology, Laboratory of Centre for Preclinical Research, Medical University of Warsaw, Warsaw, Poland; 2 Department of Neurosurgery, Mossakowski Medical Research Centre, Polish Academy of Sciences, Warsaw, Poland; 3 Mass Spectrometry Laboratory, Institute of Biochemistry and Biophysics, Polish Academy of Sciences, Warsaw, Poland

**Keywords:** Butyric acid, Blood pressure, SCFA, Bacterial metabolites

## Abstract

Butyric acid (BA) is a short-chain fatty acid (SCFA) produced by gut bacteria in the colon. We hypothesized that colon-derived BA may affect hemodynamics. Arterial blood pressure (BP) and heart rate (HR) were recorded in anesthetized, male, 14-week-old Wistar rats. A vehicle, BA, or 3-hydroxybutyrate, an antagonist of SCFA receptors GPR41/43 (ANT) were administered intravenously (IV) or into the colon (IC). Reactivity of mesenteric (MA) and gracilis muscle (GMA) arteries was tested ex vivo. The concentration of BA in stools, urine, portal, and systemic blood was measured with liquid chromatography coupled with mass spectrometry. BA administered IV decreased BP with no significant effect on HR. The ANT reduced, whereas L-NAME, a nitric oxide synthase inhibitor, did not affect the hypotensive effect of BA. In comparison to BA administered intravenously, BA administered into the colon produced a significantly longer decrease in BP and a decrease in HR, which was associated with a 2–3-fold increase in BA colon content. Subphrenic vagotomy and IC pretreatment with the ANT significantly reduced the hypotensive effect. Ex vivo, BA dilated MA and GMA. In conclusion, an increase in the concentration of BA in the colon produces a significant hypotensive effect which depends on the afferent colonic vagus nerve signaling and GPR41/43 receptors. BA seems to be one of mediators between gut microbiota and the circulatory system.

**Electronic supplementary material:**

The online version of this article (10.1007/s00424-019-02322-y) contains supplementary material, which is available to authorized users.

## Introduction

Ample evidence suggests that molecules produced by gut microbiota such as short-chain fatty acids (SCFAs) exert a significant effect on mammalian homeostasis [4, 20]. SCFAs are used by intestinal cells as an energy source. However, accumulating data imply that these molecules may also produce systemic effects [8, 19, 25, 29] acting via free fatty acid receptors GPR41, GPR43, and olfactory receptors 78 [6, 9, 14, 15, 23].

SCFAs include several carboxylic acids produced by bacterial fermentation of dietary fibers such as butyric acid (BA). There are some studies suggesting that BA affects arterial blood pressure. For example, a biphasic hemodynamic effect of intravenous administration of tributyrin, a BA prodrug, was described in 1957 by Wretlind [33]. Recently, a hypotensive effect was reported after administration of BA into the kidney medulla [31] and intraperitoneally [25] in rats. However, those studies did not evaluate BA blood level and it is difficult to speculate whether the reported effects are of physiological, pharmacological, or suprapharmacological importance.

Notably, although BA is produced in the gut, the effects of BA on the gut-circulatory system axis have not been evaluated. The latter is important as ample research shows that gut signaling plays a significant role in interactions between gut microbiota and the host [5, 27].

Finally, the colon, a major site of bacterial metabolism, expresses receptors for SCFA [13, 24, 28].

Therefore, we evaluated whether BA may exert hemodynamic effects via gut signaling. Furthermore, we aimed to establish physiological concentrations of BA in the colon content (stools), portal blood, systemic blood, and urine in rats.

## Methods

### Animals

The experiments were performed according to Directive 2010/63 EU on the protection of animals used for scientific purposes and approved by the I Local Bioethical Committee in Warsaw (permission no 534/2018 and 535/2018). Experiments were performed on 14–16-week-old, male Wistar rats (Mossakowski Medical Research Center Polish Academy of Sciences, Warsaw, Poland) fed a standard laboratory diet (Labofeed B standard, Kcynia, Poland), food and water ad libitum. Rats were housed in groups (3–4) in polypropylene cages with environmental enrichment, 12-h light/12-h dark cycle, temperature 22–23 °C, humidity 45–55%.

### Evaluation of BA levels in body fluids

Rats (*n* = 9) were maintained for 2 days in metabolism cages to evaluate 24 h water and food balance and to collect urine for BA analysis. Data from the second day were analyzed. Next, rats were anaesthetized with 15% solution of urethane (i.p. 1.5 g/kg bw, Sigma-Aldrich, Poznan, Poland) and were implanted with polyurethane catheters inserted into the portal vein and into the inferior vena cava as we previously described [10]. After the blood taking (0.25 ml each sample), rats were killed by decapitation. The evaluation of BA concentration was performed in portal and systemic blood plasma. A 7–8-cm long segment of the colon (a middle part between the cecum and the rectum) was closed with sutures and removed. A sample of 0.5 ml of stools was collected from the removed colon, weighted and homogenized with 1 ml of 0.9% NaCl in a closed 2-ml laboratory tube by vortexing it for 5 min. Afterwards, the sample was centrifuged for 5 min at 5000 rpm, and 1 ml of the obtained supernatant was transferred to a laboratory tube and again centrifuged for 5 min. All procedures were performed at the temperature of 2–5 °C. The supernatant was collected into Eppendorf tubes and frozen at − 20 °C. BA concentration in the colon content was calculated as BA concentration in the supernatant multiplied by a factor of 3 (as described above, 1 ml of saline was added to 0.5 ml of colon content to prepare supernatant for analysis).

### Hemodynamic studies

The measurements were performed under general anesthesia with 15% solution of urethane (i.p. 1.5 g/kg b.w, Sigma-Aldrich, Poznan, Poland). Before the measurements, rats were implanted with a polyurethane arterial catheter which was inserted through the femoral artery into the abdominal aorta and connected to the BP recording system, BIOPAC MP160 (Biopac Systems, Goleta, USA). For intravenous treatment, a catheter was implanted into the femoral vein. For electrocardiogram (ECG) recordings, standard needle electrodes were used (Biopac).

The measurements were started 40 min after the induction of anesthesia, and 10 min after connecting the arterial and venous catheters.

Hemodynamic studies comprised the following experimental series performed on separate groups of rats:

#### Intravenous administrations

1. Intravenous administration of a vehicle (saline 0.2 ml/2 min, *n* = 5), saline solution of BA at a dose of 0.14 (*n* = 5), 1.4 (*n* = 5), 2.8 (*n* = 5) and 5.6 mmol/kg (*n* = 5), and ANT at a dose of 5.6 mmol/kg (*n* = 5).
2. Intravenous administration of BA at a dose of 1.4 mmol/kg after the pretreatment with the ANT at a dose of 1.4 mmol/kg (*n* = 5).
3. Intravenous administration of BA at a dose of 1.4 mmol/kg after the pretreatment with L-NAME, a non-specific nitric oxide synthase inhibitor, at a dose of 0.3 mmol/kg (*n* = 5), and the administration of L-NAME alone (*n* = 5).

#### Administrations into the colon

1. Administration of a vehicle (saline, 0.25 ml/30s, *n* = 5), saline solution of BA (Sodium butyrate, Sigma-Aldrich, Poznan, Poland) at a dose of 1.4 (*n* = 5), 2.8 (*n* = 5) and 5.6 mmol/kg (*n* = 5) and ANT at a dose of 5.6 mmol/kg (*n* = 5).
2. Administration of BA at a dose of 5.6 mmol/kg after the pretreatment with the ANT (3-hydroxybutyrate, Sigma-Aldrich, Poznan, Poland) at a dose of 5.6 mmol/kg (*n* = 5).
3. Administration of BA at a dose of 5.6 mmol/kg after the subphrenical vagotomy (*n* = 5), administration of BA at a dose of 5.6 mmol/kg after the sham procedure (*n* = 5) and vagotomy alone (*n* = 5).
4. Administration of BA at a dose of 5.6 mmol/kg during the intravenous treatment with hexamethonium, an autonomic ganglia blocker (Sigma-Aldrich, Poznan, Poland, 15 mg/kg bolus followed by continuous infusion at a rate of 1.5 mg/kg/min, *n* = 5) or after the intravenous treatment with atropine (Atropinum Sulfuricum WZF, Polfa Warszawa, Warsaw, Poland) at a dose of 1 mg/kg (*n* = 5).

After hemodynamic studies, rats were killed by decapitation.

#### Vagotomy

The surgical vagotomy, i.e., bilateral abdominal (subdiaphragmatic) truncal vagotomy, was performed as previously described [2] with some modification. In short, after cutting skin and muscles from the xiphoid to the navel, the liver was stabilized with ligatures to visualize the subdiaphragmatic vagus nerves. Saline solution of methylene blue (0.4%) was applied on tissues for better visualization of nerves. The nerves were cut with vascular scissors. After the procedure, the wound was stitched. The sham procedure followed the same steps except that the nerves were not cut.

It needs to be mentioned that in contrast to humans, in rats, all the regions of the colon, except the rectum, are innervated by the branches of the vagus nerve [3].

#### Intracolonic administrations

The intracolonic infusions were performed by means of a pediatric Foley catheter (10F) inserted into the colon, 8 cm from the anus as we previously described [10].

### Changes in BA concentration in stools, portal, and systemic blood after the intracolonic (IC) administration of BA

BA concentration in the colon content (stools) was evaluated in control rats (*n* = 5) and rats administered with BA into the colon at a dose of 2.8 mmol/kg (*n* = 6), as we described above. To evaluate portal and systemic blood plasma concentration of BA, blood samples (0.25 ml) from the portal vein and vena cava were collected at baseline and 20 and 60 min after the IC administration of BA at a dose of 2.8 mmol/kg (*n* = 6). The selected time points corresponded to the maximal hypotensive effect and return of BP to baseline according to our findings from the hemodynamic part of the study.

### Ex vivo reactivity studies

#### Isolation of mesenteric (MA) and gracilis muscle arteries (GMA)

Rats (*n* = 11) were anesthetized with an intraperitoneal injection of 15% solution of urethane (1.5 g/kg b.w). The mesenteric and gracilis muscle arterial bed was dissected and placed in a petri dish filled with cold (4 °C, pH = 7.4) physiological saline buffered with MOPS (3-(*N*-morpholino) propanesulfonic acid) (MOPS-PSS) containing: 3.0 mM MOPS, 144.0 mM NaCl, 3.0 mM KCl, 2.5 mM CaCl_2_, 1.5 mM MgSO_4_, 1.21 mM NaH_2_PO_4_, 0.02 mM EDTA, 2.0 mM sodium pyruvate, 5.0 mM glucose, and 1% dialyzed bovine serum albumin (BSA). The branches of the mesenteric artery (MA, 250–370 μm diameter) and gracilis muscle artery (GMA, 225–280 μm diameter) were carefully cleaned of surrounding tissues under a dissecting microscope (SZ51, Olympus, Germany) and transferred to an organ chamber. After cannulation of one end of the vessel, the blood from the lumen was gently removed, and then the other end of the vessel was mounted on the distal pipette, and both ends were secured with a 10–0 nylon suture. The organ chamber was placed on the stage of an inverted microscope (CKX41, Olympus, Germany) equipped with a video camera and a monitor. The transmural pressure was set at 50 mmHg for the MA and 80 mmHg for the GMA. The experiments were performed without intraluminal flow. The extraluminal fluid was switched to MOPS-PSS without BSA, slowly heated to 37 °C and exchanged at a rate of 20 mL/min with the help of a peristaltic pump (Masterflex, Cole-Parmer, USA).

#### Experimental protocol

After 60 min equilibration at 37 °C, the arteries were pre-constricted with phenylephrine (PE, 0.5 μM), and after the contraction reached a steady state, acetylcholine (ACh, 1 μM) was added to MOPS-PSS. Arteries which relaxed in response to ACh by more than 90% were considered as endothelium intact vessels.

The responses to increasing concentrations of butyric acid (BA, starting from 5 μM up to 1 mM) were studied in pre-constricted GMA and MA branches. The starting point was equal to a physiological blood concentration of BA in the rat.

In separate series of experiments, the ANT was administered in increasing concentrations equimolar to BA (from 5 μM to 1 mM) with BA (5 μM) in the background.

In conclusion of the experiments with ANT, the response to 1 mM BA was studied to verify whether ANT (1 mM) affects the vasorelaxant effect of BA at this concentration. Only one experimental protocol was carried out on the same vessel.

The effects of each concentration of the tested substances on the inner diameter of MA branches and GMA were assessed 15 min after their administration. At the end of each experiment, the MOPS-PSS bath solution was replaced with Ca^2+^-free PSS (PSS containing 3 mM EGTA) in which the vessels were incubated for 15 min to determine maximal passive diameter.

All values are expressed as means ± SE. Vasodilatation, as percent of the maximal diameter, was calculated based on a formula (D_active_ − D_baseline_)/(D_passive_ − D_baseline_) × 100%, where D_active_ is the measured diameter for a given dose of the tested compound, D_baseline_ is the baseline diameter measured before administration of the drug, and D_passive_ is the maximal passive diameter.

### BA concentration analysis

BA concentration analysis was performed using Waters Acquity Ultra Performance Liquid Chromatograph coupled with Waters TQ-S triple-quadrupole mass spectrometer. For the instrument control and data acquisition, Waters MassLynx software was used. Waters TargetLynx was used to processed data. LC/MS/MS analysis was performed in negative electrospray ionization mode (ESI). The mass spectrometer operated in multiple-reaction monitoring (MRM). The analytes were separated using a Waters BEH C18 column (1.7 μm, 2.1 mm × 50 mm) and Waters BEH C18 guard column (1.7 μm, 2.1 mm × 5 mm). Mobile phase A consisted of 1 mL of formic acid in 1 L water, and mobile phase B consisted of 1 mL of formic acid in acetonitrile. The flow rate of mobile phase was set at 0.6 mL/min.

Sample preparation was as follows: 80 μL methanol (containing internal standards) was added to 40 μL of sample (plasma, stool extract, urine and calibrators). After vortexing, 20 μL of 3NPH solution and 20 μL of EDC-pyridine solution were added and the mixture was incubated in room temperature for 30 min. The solution was diluted to 1 mL with 15% aqueous acetonitrile, centrifuged, and aliquot was injected into the apparatus.

To define the relationship between the concentration and detector response for analytes, calibration points were prepared. Calibration curves for BA were generated by comparing a ratio of the peak area of the analyzed compound to the peak of the corresponding internal standard against known analyte concentrations. The limits of quantification (LOQ) were 1 μM for BA.

### Chemicals

Pyridine anhydrous, 3-nitrophenylhydrazine (3NPH·HCl), and *N*-(3-dimethylaminopropyl)-*N*′-ethylcarbodiimide (EDC·HCl) were acquired from Sigma-Aldrich (St. Louis, MO, USA). LC-MS grade acetonitrile, HPLC grade acetonitrile, HPLC grade methanol, and formic acid were obtained from J.T. Baker. Ultra-pure water (Mili-Q water) was produced by a water purification system (Mili-Q, Millipore, Milford, MA, USA).

Urethane, DMSO, BA, MOPS-PSS, BSA, ANT-3-hydroxybutyrate, hexamethonium, and all reagents used to study isolated vessels were purchased from Sigma-Aldrich, Poznan, Poland

### Data analysis and statistics

Mean arterial blood pressure (MABP) and heart rate (HR) were calculated on blood pressure tracing by Acq Knowledge software (Biopac Systems, Goleta, USA). To evaluate ECG, lead II was used. The length of QT was manually measured from the onset of QRS complex to the end of T wave. The average of 10 consecutive QT intervals was used for analysis. Corrected QT (QTc) was calculated according to the following formula: QTc = QT/(RR/f)1/2, where f is the normalization factor according to the basal RR interval duration in rats (150 ms) [17]. For the evaluation of changes in hemodynamic parameters and SCFA blood level in response to the treatment, baseline values were compared with values after the treatment by means of one-way analysis of variance (ANOVA) for repeated measures, followed by Tukey’s post hoc test. Differences between the groups/series were evaluated by multivariate ANOVA, followed by Tukey’s post hoc test or by *t* test, when appropriate. The Kolmogorov-Smirnov test was used to test normality of the distribution. A value of two-sided *p* < 0.05 was considered significant. Analyses were conducted using Dell Statistica, version 13 (Dell Inc, Tulsa, USA).

## Results

### Physiological levels of BA in body fluids in rats

Basic metabolic parameters and concentration of BA in the colon content, portal blood plasma, systemic blood plasma, and urine are presented in Table 1. The concentration of BA was the highest in the colon content ≈ 8 mM (Table 1). Portal blood concentration of BA was approximately two orders of magnitude lower whereas systemic blood concentration of BA was approximately three orders of magnitude lower than the concentration of BA in the colon content.

**Table 1:** Basic metabolic parameters, concentration of butyric acid (BA) in the colon content, portal blood, systemic blood plasma, urine (μM), and daily urine excretion (μmol/24 h) of BA in Wistar rats (*n* = 9). Presented data are means ± SE

General metabolic data	
Body weight (g)	314.8 ± 25.2
Food intake (g)	24.3 ± 2.6
Water intake (ml)	35.6 ± 4.3
24-h urine output (ml)	18.8 ± 5.0
Stools output (g)	10.3 ± 2.1
BA concentrations/excretion	
Colon content (μM)	7,992 ± 445
Portal blood (μM)	129 ± 37
Systemic blood (μM)	4.25 ± 1.26
Urine (μM)	15.6 ± 3.89
Daily urine excretion (μmol/24 h)	0.294 ± 0.073

### BA administered intravenously produced a transient decrease in BP

There were no significant differences in hemodynamic parameters between experimental series at baseline (Table 2).

**Table 2:** Baseline mean arterial blood pressure (MABP, mmHg) and heart rate (HR, beats/min) in Wistar rats. Means ± SE are presented

Series	MABP	HR
Vehicle	100.5 ± 1.3	364 ± 14
ANT	101.8 ± 2.1	346 ± 11
BA 5.6 mmol/kg	100.9 ± 5.2	351 ± 11
BA 2.8 mmol/kg	103.2 ± 0.5	331 ± 16
BA 1.4 mmol/kg	95.2 ± 4.7	336 ± 20
BA 0.14 mmol/kg	107.2 ± 3.2	385 ± 12
BA + ANT	101.1 ± 1.3	362 ± 11
BA + L-NAME	99.4 ± 7.3	371 ± 11
L-NAME	99.5 ± 5.6	349 ± 17

**Fig. 1:**
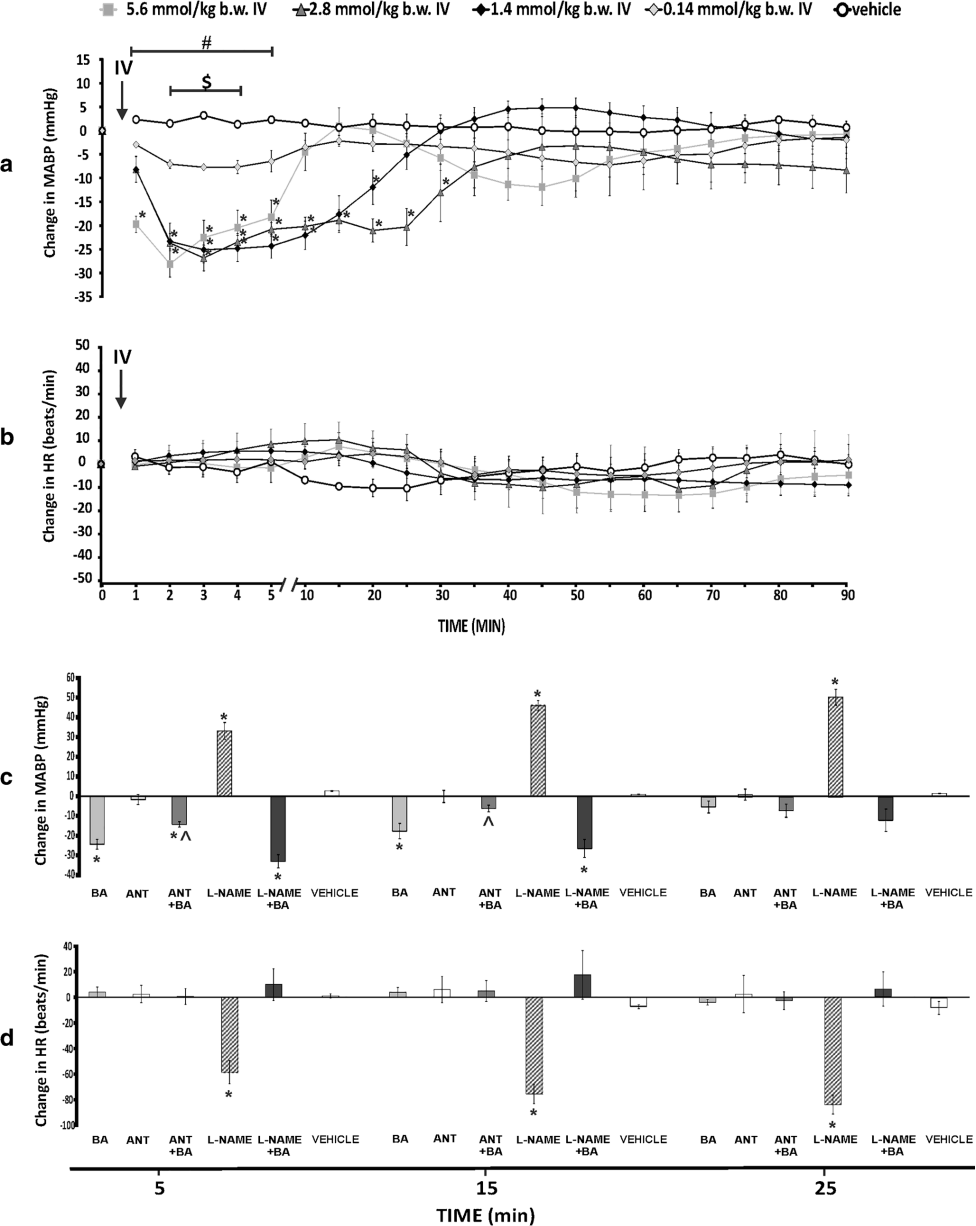
**a** Changes in mean arterial blood pressure (ΔMABP, mmHg) and **b** heart rate (ΔHR, beats/min) in Wistar rats after the intravenous administration (IV) of either a vehicle (0.9% NaCl) or butyric acid (BA) at a dose of 0.14, 1.4, 2.8, and 5.6 mmol/kg; *p < 0.05 vs baseline, ^**#**^*p* < 0.05: 1.4, 2.8, and 5.6 mmol/kg BA series vs the vehicle, ^**$**^*p* < 0.05: 0.14 mmol/kg BA series vs 2.8 and 5.6 mmol/kg BA series. c, d ΔMABP and ΔHR after the intravenous infusions of BA at a dose of 1.4 mmol/kg, (BA) or 3-hydroxybutyrate, a non-specific antagonist of GPR41/43 receptors at a dose of 1.4 mmol/kg (ANT), or BA after the pretreatment with ANT (ANT + BA) or a non-specific nitric oxide synthase inhibitor at a dose of 0.3 mmol/kg (L-NAME) or the administration of BA after the pretreatment with L-NAME (L-NAME + BA), or the vehicle. *p < 0.05 vs baseline, **^***p* < 0.05 vs BA series. Means ± SE are presented

BA at a dose of 1.4, 2.8, and 5.8 mmol/kg produced a significant, transient decrease in MABP (Fig. 1). The hypotensive response was not associated with significant changes in HR (Fig. 1). Administration of the vehicle and ANT did not produce a significant change in MABP and HR (Fig. 1).

Pretreatment with L-NAME did not affect significantly hypotensive effect of BA, whereas pretreatment with ANT moderately reduced the hypotensive effect of BA (Fig. 1 and Figs. S1–S4).

### BA administered into the colon produced prolonged decrease in BP and HR

There were no significant differences in hemodynamic parameters between experimental series at baseline (Table 3).

**Table 3:** Baseline mean arterial blood pressure (MABP, mmHg) and heart rate (HR, beats/min) in Wistar rats. Means ± SE are presented

Series	MABP	HR
Vehicle	98.4 ± 1.8	350 ± 16
ANT	96.1 ± 1.9	346 ± 13
BA 5.6 mmol/kg	102.3 ± 3.4	352 ± 13
BA 2.8 mmol/kg	104.1 ± 1.4	355 ± 14
BA 1.4 mmol/kg	98.9 ± 6.3	343 ± 11
BA + ANT	99.3 ± 2.4	321 ± 17
BA + vagotomy	98.6 ± 2.2	357 ± 31
BA + sham vagotomy	95.5 ± 4.3	313 ± 37
Vagotomy	97.9 ± 5.4	349 ± 21
BA + Hexamethonium	100.3 ± 4.5/64.8 ± 2.4^a^	365 ± 16/268 ± 3^a^
BA + Atropine	95.2 ± 2.6/90.7 ± 3.4^b^	376 ± 8/365 ± 5^b^

^a^After the pretreatment with hexamethonium but before administration of BA

^b^After the pretreatment with atropine but before administration of BA

**Fig. 2:**
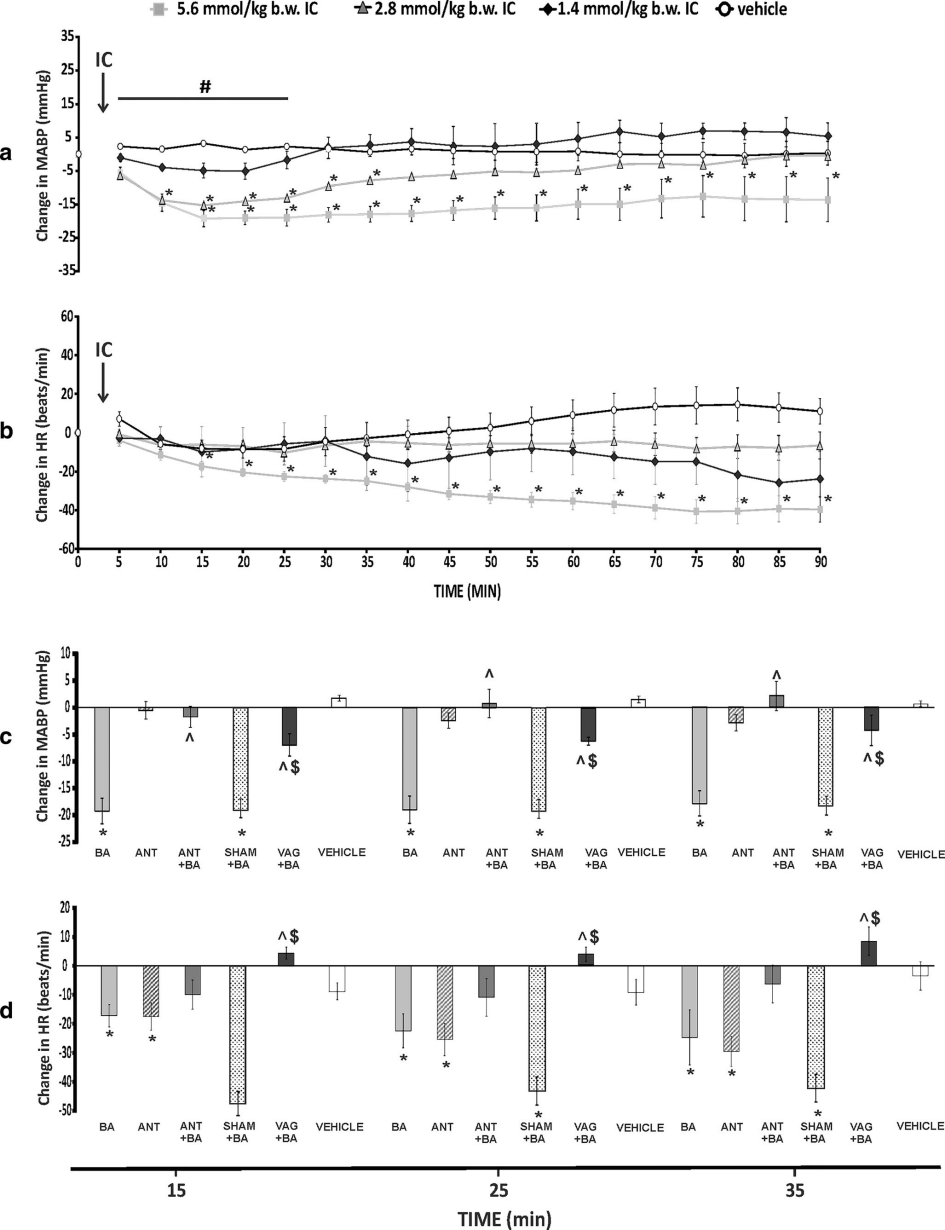
**a** Changes in mean arterial blood pressure (ΔMABP, mmHg) and **b** heart rate (ΔHR, beats/min) in Wistar rats after the intracolonic administration (IC) of either a vehicle (0.9% NaCl) or butyric acid (BA) at a dose of 1.4, 2.8, and 5.6 mmol/kg. *p < 0.05 vs baseline, ^**#**^*p* < 0.05 - 2.8 and 5.6 mmol/kg BA series vs the vehicle. **c**, **d** ΔMABP and ΔHR after the IC administration of BA at a dose of 5.6 mmol/kg (BA), or 3-hydroxybutyrate, a non-specific antagonist of GPR41/43 receptors at a dose of 5.6 mmol/kg (ANT) or BA after the pretreatment with ANT (ANT + BA), or the administration of BA after the sham vagotomy (SHAM + BA) or the administration of BA after the vagotomy (VAG + BA), or the vehicle. *p < 0.05 vs baseline, **^***p* < 0.05 vs BA series, ^**$**^*p* < 0.05 vs SHAM + BA series. Means ± SE are presented

BA administered IC produced a dose-dependent decrease in MABP, i.e., 1.4 mmol/kg series showed mild and not significant decrease, whereas 2.8 and 5.6 mmol series showed a significant decrease in MABP which lasted throughout the experiment (90 min) for 5.6 mmol/kg series (Fig. 2). This was associated with a significant decrease in HR in 5.6 mmol/kg series (Fig. 2), but no significant change in QTc, a marker of drug cardiotoxicity (Table 4).

**Table 4:** Electrocardiographic parameters in rats (*n* = 5) at baseline, 20 and 60 min after the intracolonic administration of BA (IC BA) at a dose of 5.6 mmol/kg. Means ± SE are presented

ECG parameters	Baseline	20 min after IC BA	60 min after IC BA
RR (ms)	182.2 ± 14.6	229.3 ± 20.8*	210.5 ± 16.3*
QT (ms)	89.1 ± 5.4	100.6 ± 3.2	102.2 ± 3.7
QTc (ms)	80.9 ± 2.5	82.0 ± 3.7	86.7 ± 3.1
PR (ms)	65.8 ± 2.2	66.2 ± 1.7	70.4 ± 0.8
QRS width (ms)	17.7 ± 0.6	19.1 ± 0.9	18.2 ± 0.2
QRS amplitude (mV)	2.9 ± 0.3	2.8 ± 0.3	2.8 ± 0.3

*p < 0.05 vs baseline

The IC pretreatment with the ANT significantly reduced the hypotensive effect of BA (Fig. 2 and Fig. S9).

The hypotensive and bradycardic response to BA was significantly reduced by subphrenic vagotomy. Namely, vagotomized rats showed significantly smaller decrease in MABP and HR than sham series after the IC administration of BA (Fig. 2 and Figs. S11 and S12). Likewise, the pretreated with hexamethonium, the autonomic ganglia blocker inhibited the hypotensive response (Fig. S13). In contrast, the pretreatment with atropine, an antagonist of acetylcholine muscarinic receptors, did not affect significantly the hypotensive response to the IC administration of BA (Fig. S10).

The vehicle, the ANT, and vagotomy alone did not affect MABP significantly (Fig. 2 and Figs. S8 and S10). The ANT did not affect significantly MABP, but produced a decrease in HR (Fig. 2 and Fig. S8).

### Changes in BA level in portal and systemic blood after administration of BA into the colon

Measurements of the concentration of BA in the colon content and in the portal and systemic blood showed that the lowest effective dose of BA which decreased arterial blood pressure increased colon content of BA by 2–3-fold, portal blood concentration of BA by 5-fold, and systemic blood concentration of BA by 20-fold (Table 5).

**Table 5:** BA concentration in portal blood and systemic blood at baseline, 20 and 60 min after the intracolonic administration (IC) of BA at a dose of 2.8 mmol/kg (*n* = 6). The selected time points correspond to the maximal hypotensive effect and return of BP to baseline according to our findings from the hemodynamic part of the study. Means ± SE are presented

BA (μM) concentration before (baseline) and after BA administration into the colon	
Stools (colon content)	
Colon content	9202 ± 1723
20 min after administration	24,128 ± 2158
Portal vein blood	
Baseline	96.9 ± 19.4
20 min after administration	528.0 ± 75.2
60 min after administration	296.9 ± 40.1
Systemic vein blood	
Baseline	4.35 ± 0.42
20 min after administration	107.5 ± 19.3
60 min after administration	43.9 ± 6.6

### BA dilated mesenteric and gracilis muscle arteries (ex vivo reactivity studies)

At 50 mmHg, the mean internal diameter of MA branches was 305 ± 12 μm (*n* = 10) and at 80 mmHg, the mean internal diameter of GMA was 245 ± 7 μm (*n* = 10). All arteries were pre-constricted with phenylephrine (0.5 μM), which decreased vessels diameter by around 50%.

#### Effect of BA on MA branches and GMA diameter

**Fig. 3:**
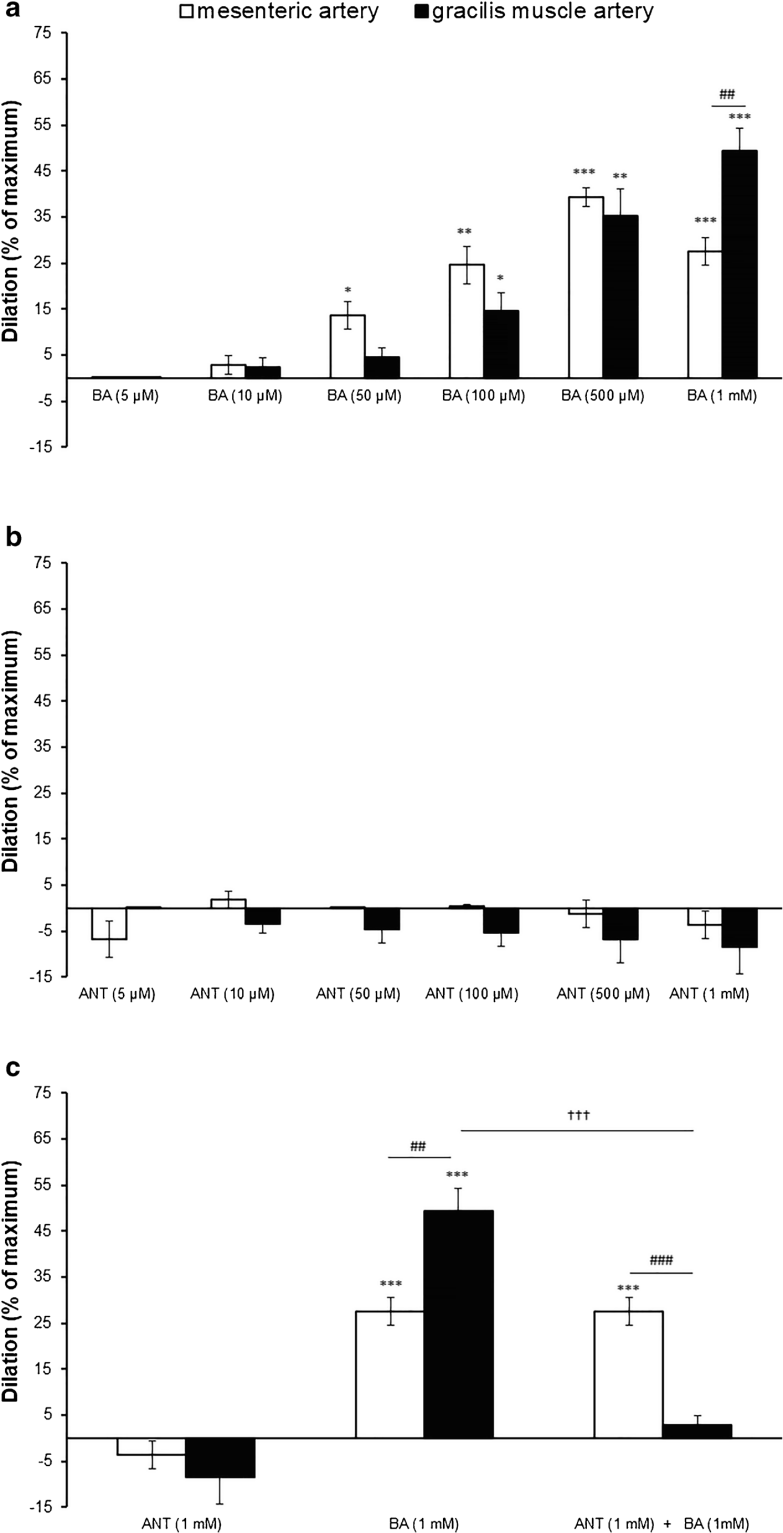
Response of the pre-constricted with PE mesenteric artery branches and gracilis muscle arteries to **a** increasing concentration of butyric acid (BA, from 5 μM up to 1 mM, *n* = 10); **b** increasing concentration of 3-hydroxybutyrate (ANT, from 5 μM to 1 mM, *n* = 10) administered concurrently with BA (5 μM), and **c** the effect of 3-hydroxybutyrate (ANT, 1 mM) administered concurrently with BA on vasorelaxant responses of mesenteric artery branches (*n* = 5) and gracilis muscle artery (*n* = 5) to BA (1 mM). Dilation is expressed as a percentage of maximum diameter (0 Ca^2+^, EGTA 3 mM). Values are means ± SE of *n* arteries. *p < 0.05, **p < 0.01, ***p, *p* < 0.001: a significant vasorelaxation; ##*p* < 0.01, ###*p* < 0.001: a significant difference between the MA vs GMA response to the BA or ANT ; †††*p* < 0.001 a significant effect of ANT (1 mM) on the vasodilation evoked by BA (1 mM)

Administration of BA at a dose equal to the physiological concentration of this acid in the systemic blood (5 μM) did not change the diameter of MA or GMA. A significant relaxation of MA by 14 ± 3% was observed at the threshold concentration of 50 μM. The response of the GMA to BA was shifted to the right, i.e., threshold concentration which resulted in increase of the GMA diameter by 15 ± 4% amounted to 100 μM. There were no significant differences in the BA response between MA and GMA, except for the last tested dose (1 mM) at which GMA responded more strongly than MA (49 ± 5% vs 28 ± 3%, respectively) (Fig. 3a).

#### Effect of 3-hydroxybutyrate (ANT) on MA branches and GMA diameter

ANT regardless of the applied concentration did not affect the MA and GMA diameters (Fig. 3b). The ANT did not affect the vasorelaxant effect of BA (1 mM) in MA branches. In contrast, ANT abolished GMA relaxation produced by BA,(Fig. 3c).

## Discussion

A new finding of our study is that BA at a dose which increases the concentration of BA in the colon by 2–3-fold exerts a significant hypotensive effect. The hypotensive effect seems to involve nervous control of arterial blood pressure including afferent colonic vagus nerve signaling and SCFA receptors GPR41/43.

Gut bacteria and their metabolites including hydrogen sulfide, indoles, and SCFA may influence the circulatory and the nervous system functions [10, 11, 14]. The mechanisms of such interaction are not clear. Nevertheless, the following two pathways are possible. Firstly, bacterial metabolites may stimulate sensory fibers of the enteric nervous system which communicate with the central nervous system [5]. Secondly, gut bacteria-derived molecules after entering the systemic circulation may reach virtually all organs involved in arterial blood pressure control.

Our study shows that physiological concentration of BA in the colon is three orders of magnitude higher than that in systemic blood, which makes the colon a very likely site of BA action. Furthermore, BA administered into the colon produced a significant hypotensive effect which was diminished by the subphrenic vagotomy and intracolonic pretreatment with a non-specific antagonist of GPR41/43. These findings suggest that the afferent arm of the hypotensive response involves colonic afferent vagus nerve signaling and GPR41/43 receptors. In this regard, the afferent fibers of the vagus nerve are known to modulate the brain centers involved in the control on the autonomic nervous system activity and blood pressure [7]. Notably, the hypotensive effect of BA administered into the colon was associated with only 2–3-fold increase in the colon concentration of BA, suggesting that such effect may be of physiological importance.

We would speculate that the hypotensive response to BA may be mediated by the inhibitory effect of BA on tonic sympathetic activity, as hexamethonium, an autonomic ganglia blocker, but not atropine, an antagonist of the acetylcholine receptors reduced the hypotensive effect of BA. The hypotensive response may also depend on a direct vasodilatory action of BA. Namely, in ex vivo studies, we found that BA produced a significant, dose-dependent vasodilation in MA and GMA.

Interestingly, in GMA, the vasodilatory effect of BA was reduced by the ANT whereas the ANT did not affect significantly the BA-induced vasodilation in MA, suggesting that the mechanisms of relaxation to BA in these two vessels differ. Although both MA and GMA are resistance vessels, they differ in the structure of the internal elastic lamina [16] and in the response to TRP agonists [30]. Our findings suggest that BA-induced vasorelaxation is dependent on GPR41/43 receptors in GMA but not in MA. It seems that the vasorelaxant effect of BA in the latter may be mediated by other than GPR41/43 receptors or may be dependent on a direct relaxation of vascular smooth muscles as postulated by Aronson and collaborators [1]. Although the relaxation of various resistance blood vessels in response to other SCFA is well documented, the underlying mechanisms are complex and not fully elucidated. Previously, Mortensen et al. showed that acetic, propionic, and BA produce a concentration-dependent (0.1–30 mM) vasorelaxant effects in human colonic resistance arteries [20]. Also, a weak vasodilatory effect of BA at concentrations above 5 mM was shown in the coronary arteries [12]. The vasodilatory effect of SCFA was also demonstrated in rat caudal artery at 0.8 mM and 1.9 mM mean effective concentrations for butyrate and propionate, respectively [21]. Several mechanisms of vasodilatory activity of BA have been proposed including stimulation of the cyclic AMP second messenger system [1] and increased synthesis of F2 alpha prostaglandins [18].

In this study, we also found a significant, hypotensive response to intravenously administered BA. The hypotensive effect of intravenously administered BA was reduced by the ANT but not by the pretreatment with L-NAME. Previously, it has been shown that BA produces relaxation of rat mesenteric arteries, which was unaffected by endothelial denudation and inhibition of NO synthase with L-NAME [1]. Altogether, it seems that hypotensive effect of BA administered intravenously was produced by vasodilation independent on nitric oxide. Interestingly, the hypotensive effect of BA in the L-NAME-treated rats was similar or even higher than in rats treated with BA alone, which points to the strong vasodilatory and hypotensive actions of BA, and perhaps reduced sympathetic component of baroreflex in the L-NAME-treated rats [26].

**Fig. 4:**
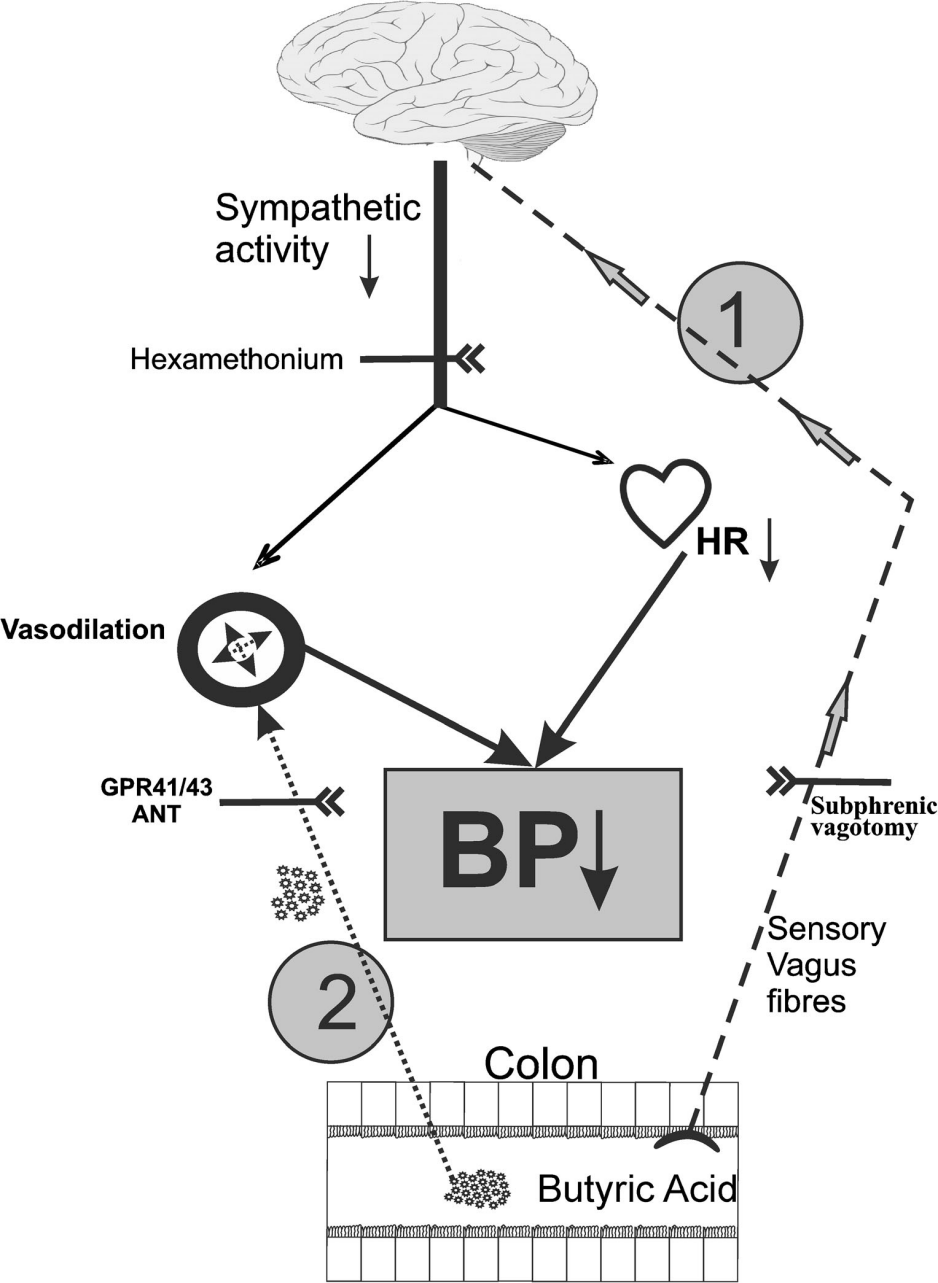
Postulated mechanisms involved in the hypotensive effect of colon-derived butyric acid (BA). **1** BA stimulates the sensory fibers of the vagus nerve that project to the brain centers controlling the circulatory system. This results in decreased tonic sympathetic activity producing a decrease in arterial blood pressure due to a decrease in HR and vasodilation. **2** BA crosses the gut-blood barrier, enters the bloodstream, and produces a direct vasodilation

Notably, the hypotensive response to BA administered intravenously was significantly shorter than the response to intracolonic administration and was not associated with HR changes. Taking together our findings, we would speculate that the hypotensive effect of intravenously administered BA was dependent on the direct BA-mediated vasodilation. In contrast, the hypotensive effect of BA administered into the colon involved also decreased sympathetic activity. The latter was produced by the afferent vagus nerve signaling from the colon to the brain (Fig. 4).

Other mechanisms that may be involved in the circulatory effects of BA but were not the focus of this study include the effect of BA on kidney [31, 32] and cardiac functions [22]. In particular, the effect of BA on diuresis needs further investigation.

A limitation of our study is that we did not evaluate the effect of Olfr78 receptor blockade, which is also thought to be involved in SCFA signaling. This is because biologically effective Olfr78 receptor blockers are not yet available. Finally, chronic interventional studies are needed to assess the effect of colonic BA on systemic blood pressure.

In conclusion, an increase in the concentration of BA in the colon by 2–3-fold exerts a significant hypotensive effect, which seems to be mediated by the colon afferent nervous signaling and GPR41/43 receptors. It may also involve vasodilation caused by blood-borne BA. Our findings provide evidence that BA is one of the mediators between gut microbiota and the circulatory system.

## Electronic supplementary material

ESM 1 (PDF 829 kb)

## Data Availability

All data generated or analyzed during this study are included in this published article (and its additional information files).

## Abbreviations

ACH: Acetylcholine
ANT: 3-hydroxybutyrate an antagonist of SCFA receptors GPR41/43
BA: Butyric acid
BP: Arterial blood pressure
BSA: Dialyzed bovine serum albumin
EDC·HCl: *N*-(3-dimethylaminopropyl)-*N*′-ethylcarbodiimide
GMA: Gracilis muscle artery
HR: Heart rate
IC: Into the colon
LOQ: Limits of quantification
MA: Mesenteric artery
MOPS-PSS: Physiological saline buffered with 3-(*N*-morpholino) propane sulfonic acid
PE: Phenylephrine
SCFA: Short-chain fatty acid
