# Supplementary material for: Butyric acid, a gut bacteria metabolite, lowers arterial blood pressure via colon-vagus nerve signaling and GPR41/43 receptors
Source: Pflugers Arch. 2019 Nov 15;471(11):1441–53. doi: 10.1007/s00424-019-02322-y (PMC6882756; doi:10.1007/s00424-019-02322-y)
Supplement: Supplementary file 1 — (PDF 829 kb) [file 424_2019_2322_MOESM1_ESM.pdf]

Supplementary Materials

Title:

Butyric acid, a gut bacteria metabolite, lowers arterial blood pressure via colon-vagus nerve signalling and GPR41/43 receptors.

INTRAVENOUS ADMINISTRATION

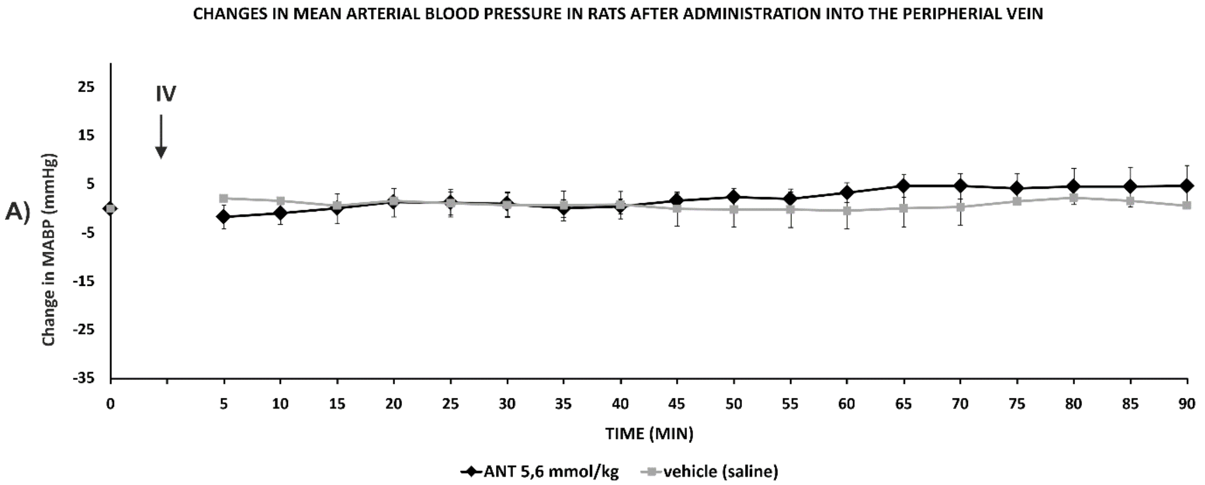

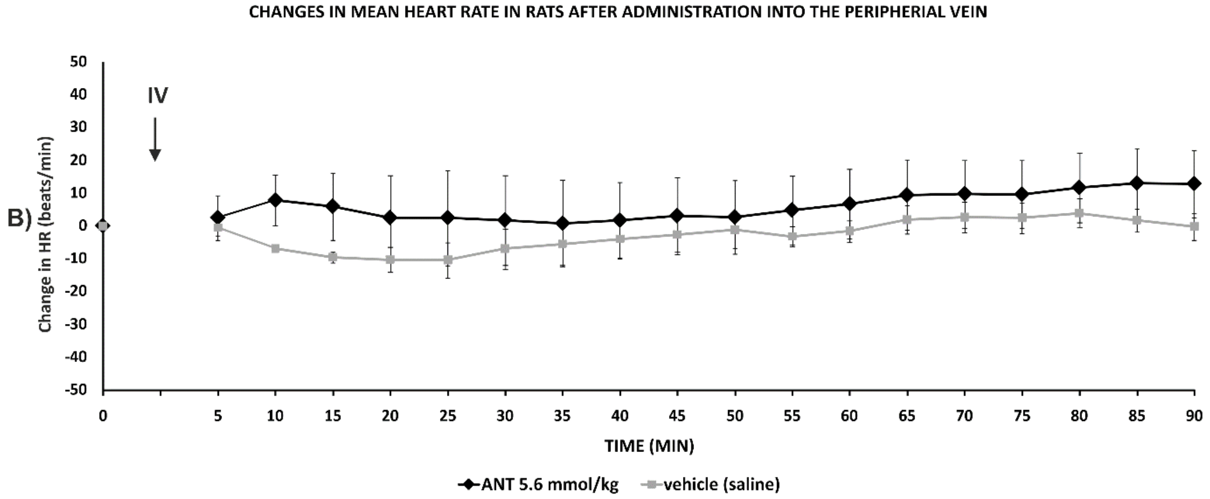

Figure S1.

[A] Changes in mean arterial blood pressure (ΔMABP, mmHg) and **[B]** heart rate (ΔHR, beats/min) in Wistar rats after the intravenous administration (IV) of either a vehicle (saline 0.2 ml/2min) or ANT at a dose of 5.6 mmol/kg. Means ± SE are presented.

* - vs Baseline

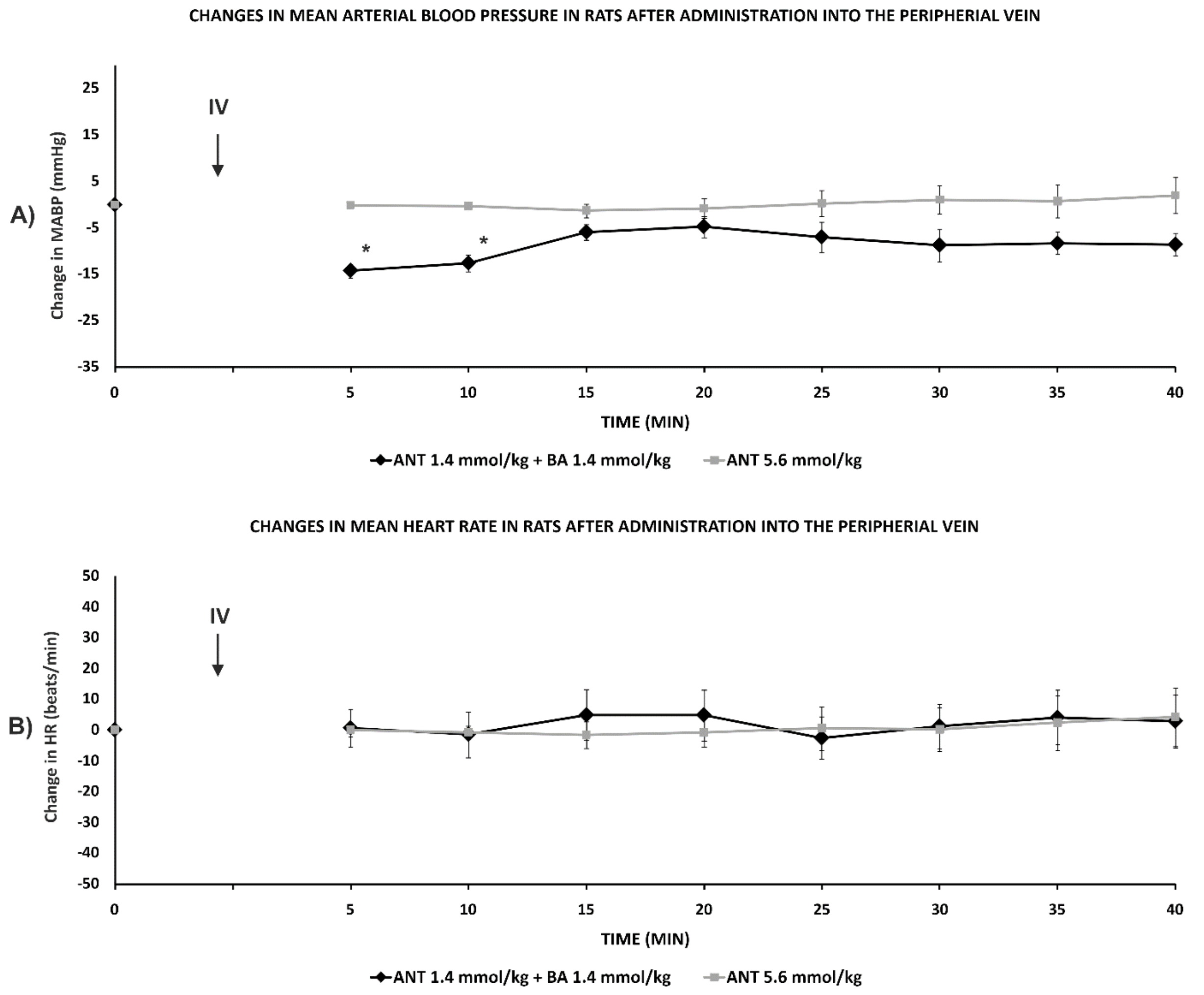

Figure S2.

[A] Changes in mean arterial blood pressure (ΔMABP, mmHg) and **[B]** heart rate (ΔHR, beats/min) in Wistar rats after the intravenous administration (IV) of BA at a dose of 1.4 mmol/kg after the pretreatment with the ANT at a dose of 1.4 mmol/kg b.w. Means ± SE are presented.

* - vs Baseline

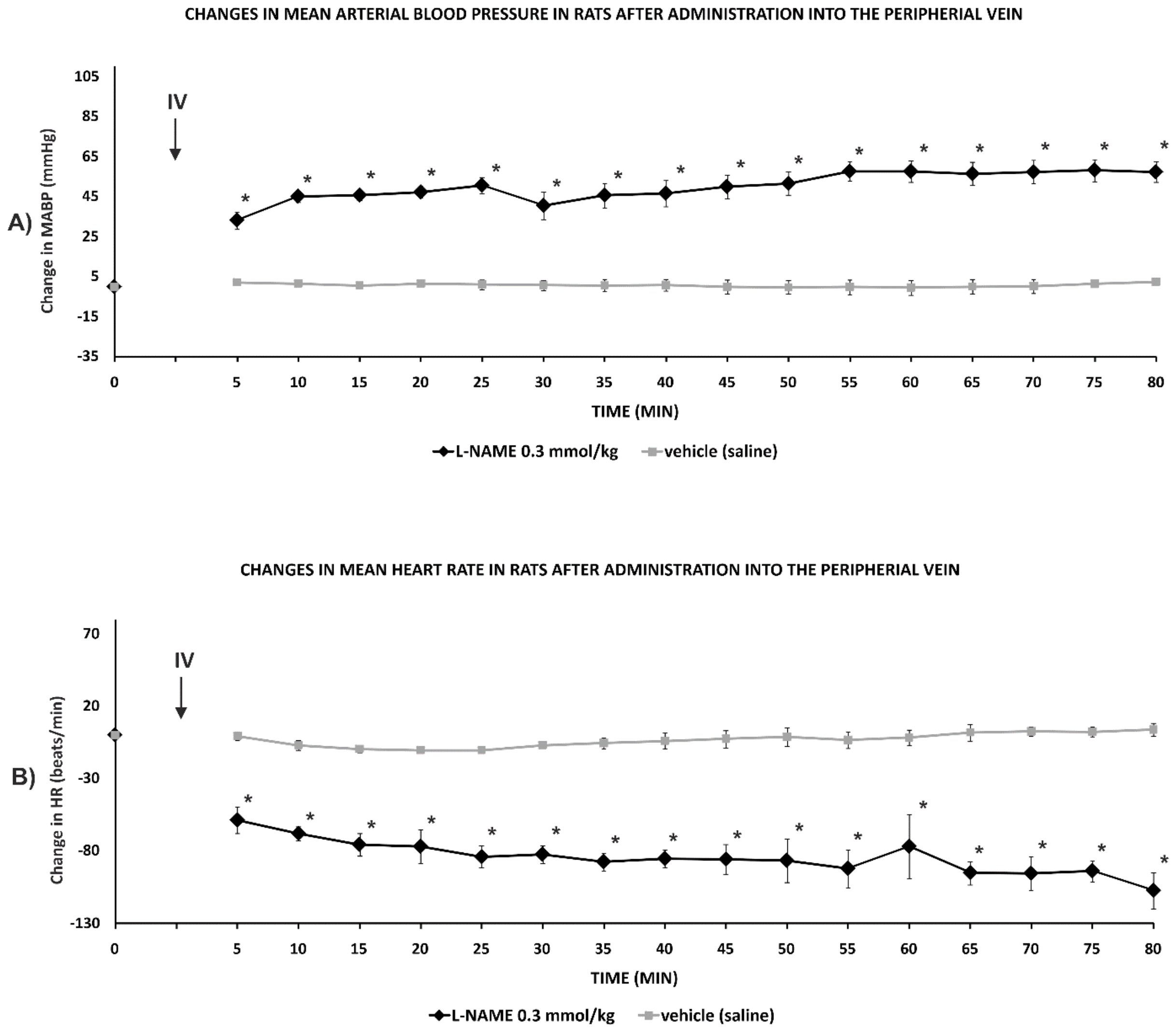

Figure S3.

[A] Changes in mean arterial blood pressure (ΔMABP, mmHg) and **[B]** heart rate (ΔHR, beats/min) in Wistar rats after the intravenous administration (IV) of L-NAME at a dose of 0.3 mmol/kg b.w. Means ± SE are presented.

* - vs Baseline

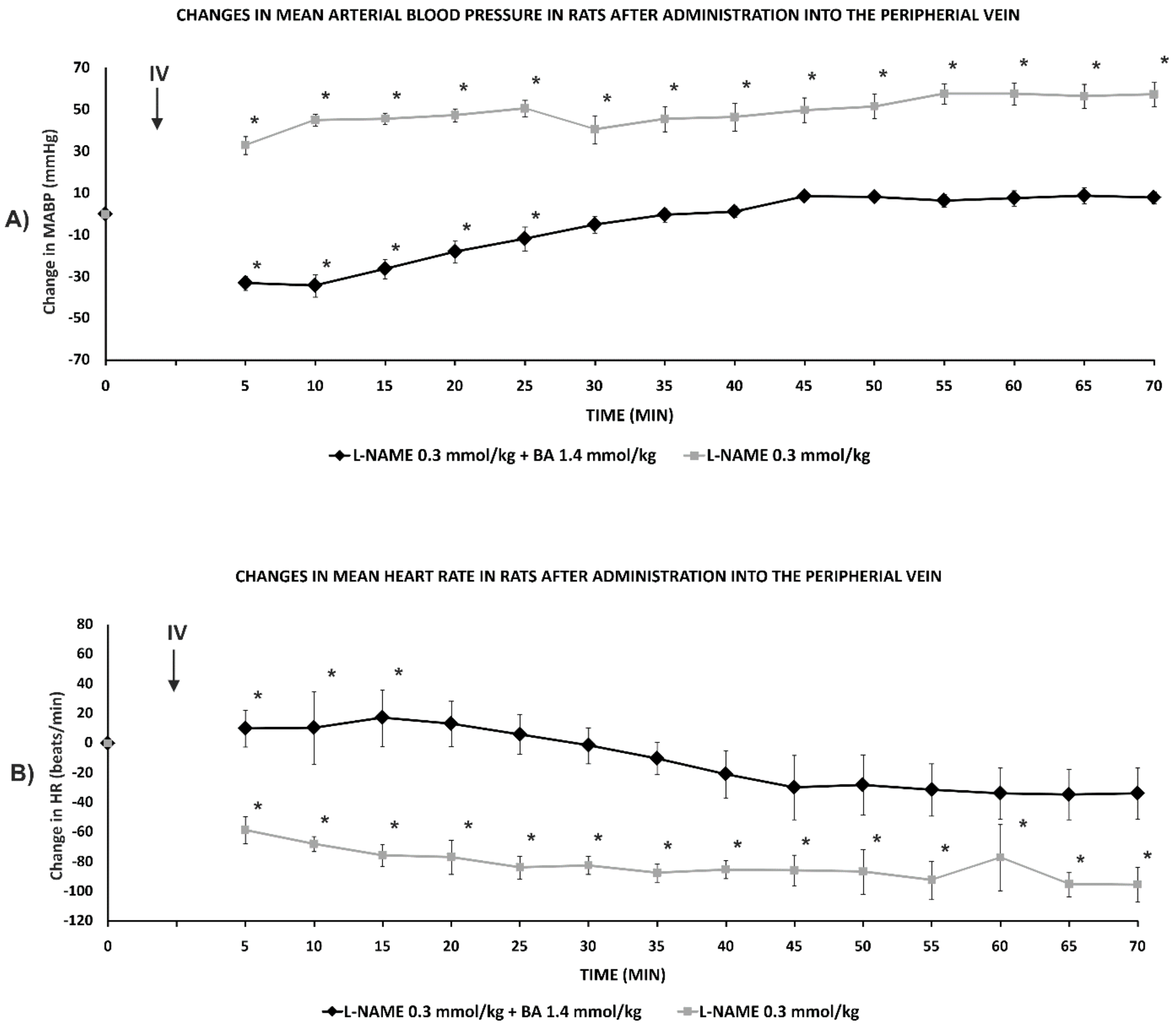

Figure S4.

[A] Changes in mean arterial blood pressure (ΔMABP, mmHg) and **[B]** heart rate (ΔHR, beats/min) in Wistar rats after the intravenous administration (IV) of BA at a dose of 1.4 mmol/kg after the pretreatment with L-NAME at a dose of 0.3 mmol/kg b.w. Means ± SE are presented.

* - vs Baseline

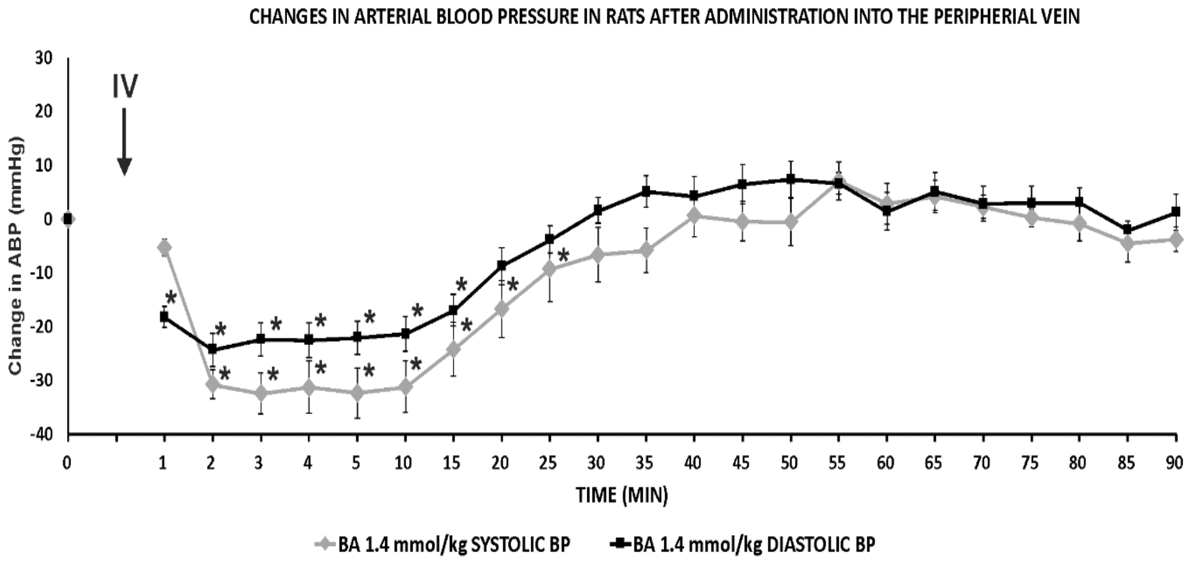

Figure S5.

Changes in arterial blood pressure (ΔABP, mmHg) in Wistar rats after the intravenous administration (IV) of BA at a dose of 1.4 mmol/kg. Means ± SE are presented.

* - vs Baseline

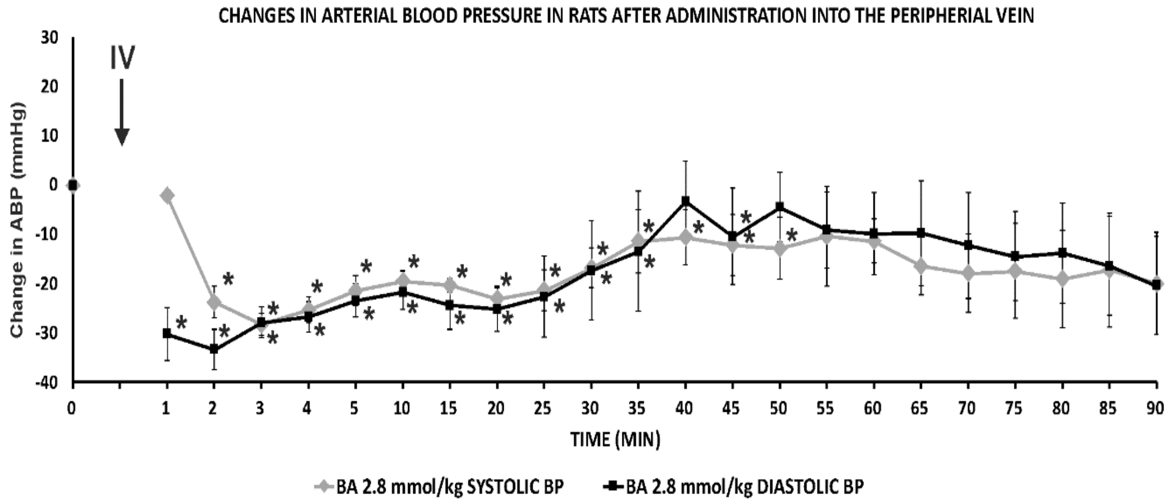

Figure S6.

Changes in arterial blood pressure (ΔABP, mmHg) in Wistar rats after the intravenous administration (IV) of BA at a dose of 2.8 mmol/kg. Means ± SE are presented.

* - vs Baseline

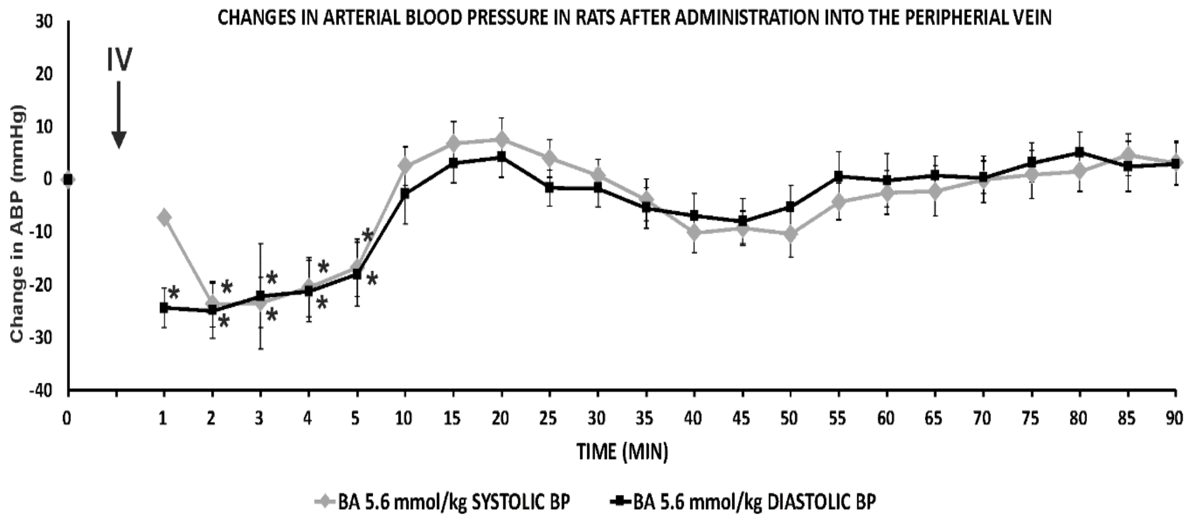

Figure S7.

Changes in arterial blood pressure (ΔABP, mmHg) in Wistar rats after the intravenous administration (IV) of BA at a dose of 5.6 mmol/kg. Means ± SE are presented.

* - vs Baseline

ADMINISTRATION INTO THE COLON

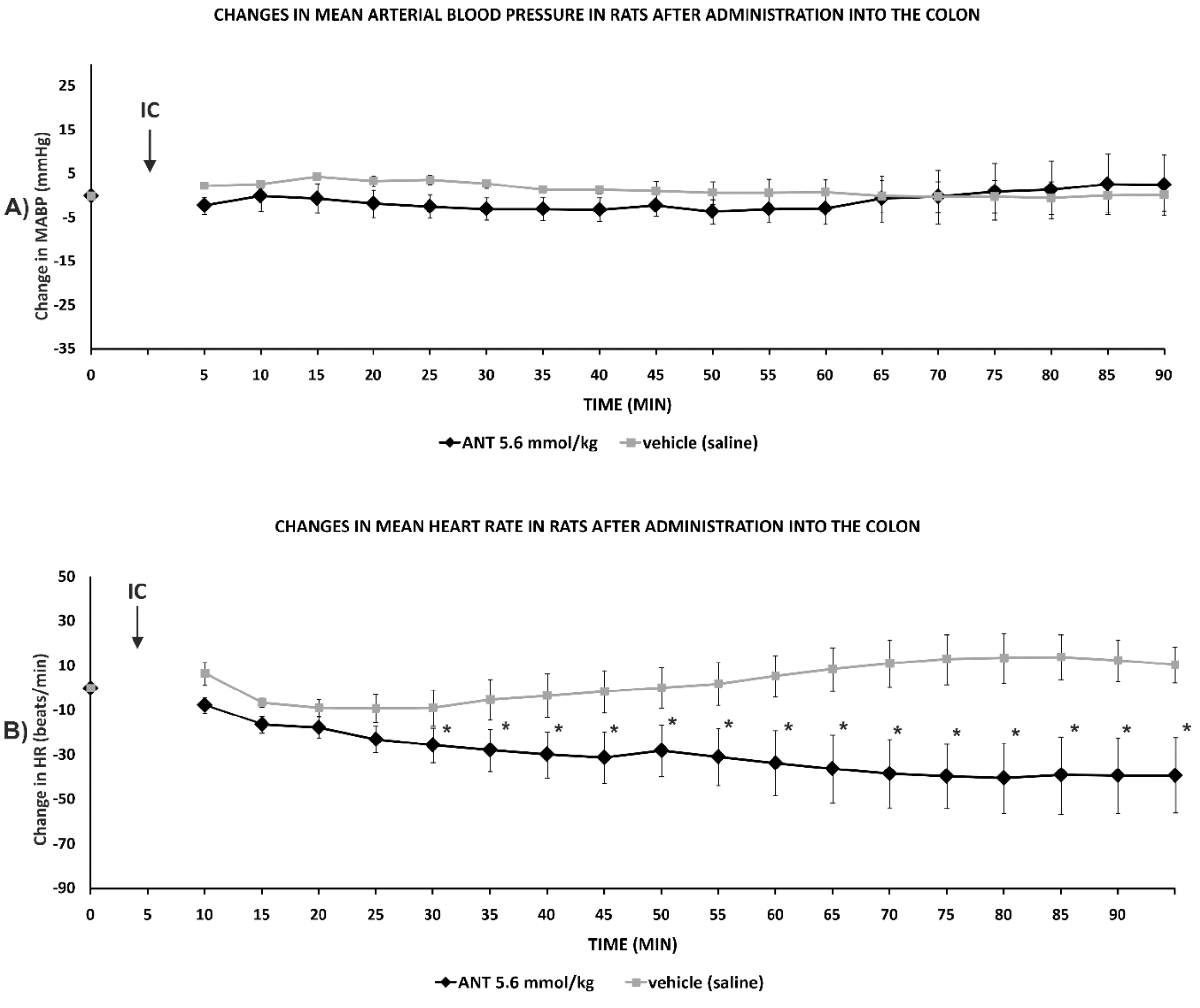

Figure S8.

[A] Changes in mean arterial blood pressure (ΔMABP, mmHg) and **[B]** heart rate (ΔHR, beats/min) in Wistar rats after administration into the colon (IC) of either a vehicle (saline, 0.25 ml/30s) or ANT at a dose of 5.6 mmol/kg b.w. Means ± SE are presented.

* - vs Baseline

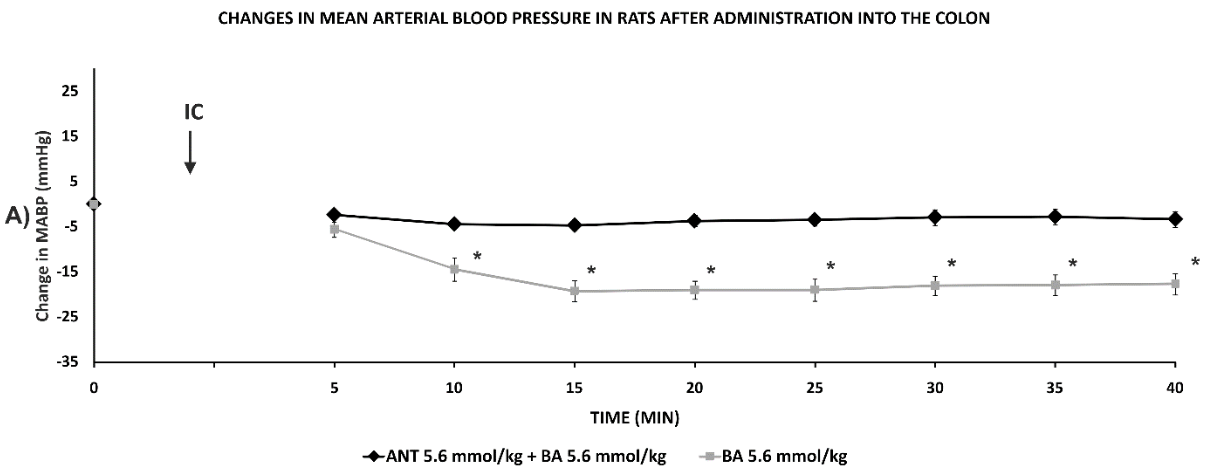

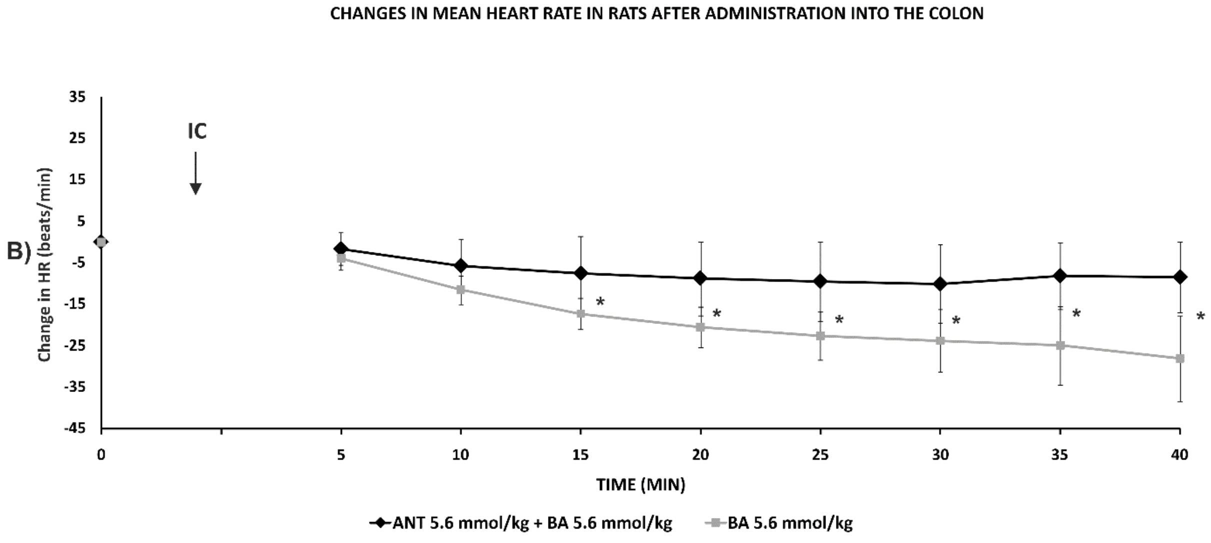

Figure S9.

[A] Changes in mean arterial blood pressure (ΔMABP, mmHg) and **[B]** heart rate (ΔHR, beats/min) in Wistar rats after administration into the colon (IC) of BA at a dose of 5.6 mmol/kg b.w. after the pretreatment with the ANT at the dose of 5.6 mol/kg b.w. Means ± SE are presented.

* - vs Baseline

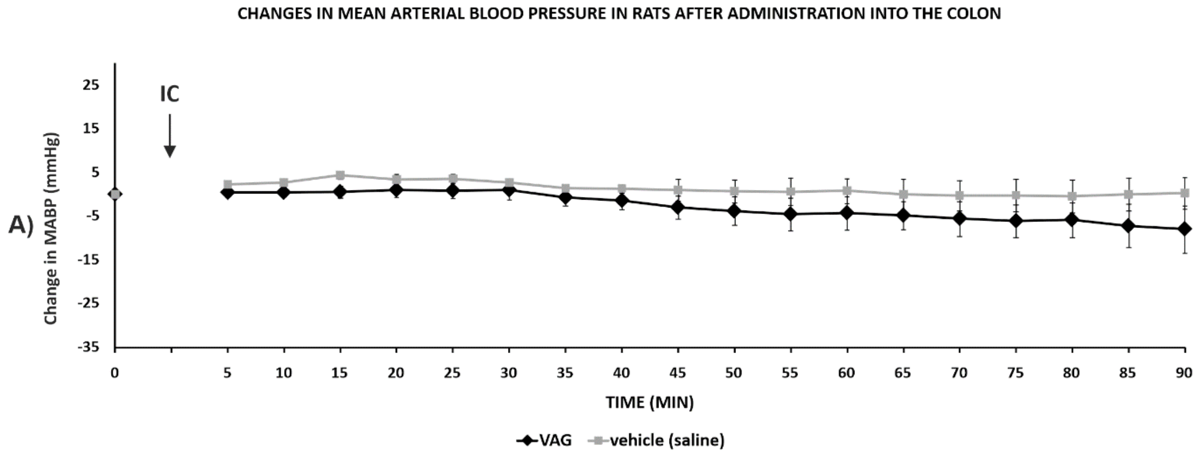

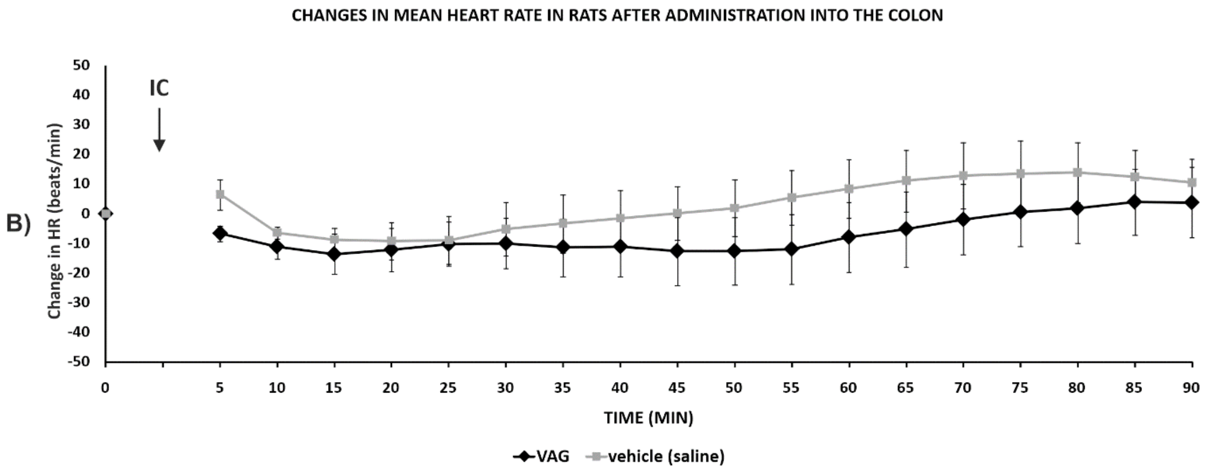

Figure S10.

[A] Changes in mean arterial blood pressure (ΔMABP, mmHg) and **[B]** heart rate (ΔHR, beats/min) in Wistar rats after the subphrenical vagotomy. Means ± SE are presented.

* - vs Baseline

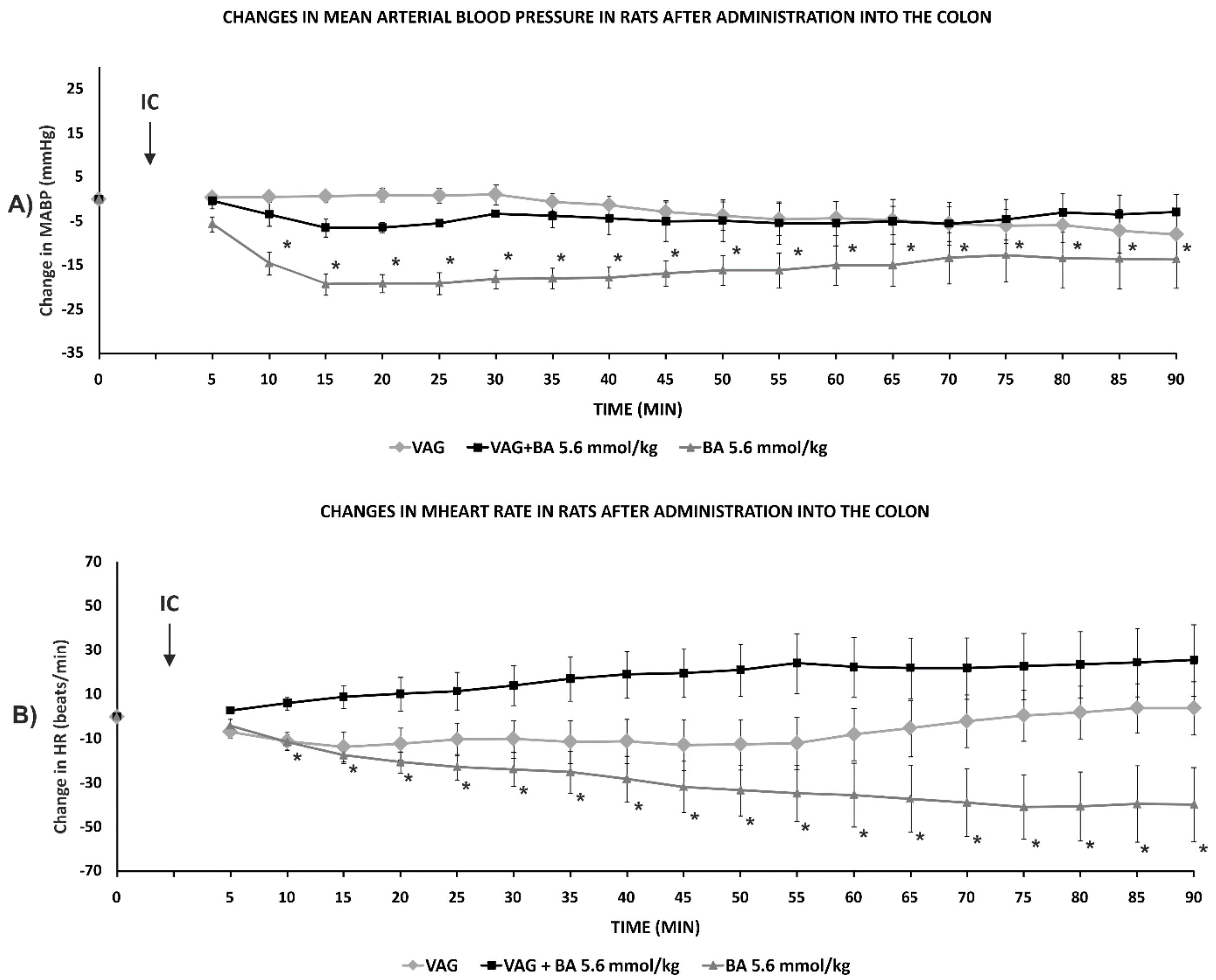

Figure S11.

[A] Changes in mean arterial blood pressure (ΔMABP, mmHg) and **[B]** heart rate (ΔHR, beats/min) in Wistar rats after administration into the colon (IC) of BA at a dose of 5.6 mmol/kg b.w. after the subphrenical vagotomy. Means ± SE are presented.

* - vs Baseline

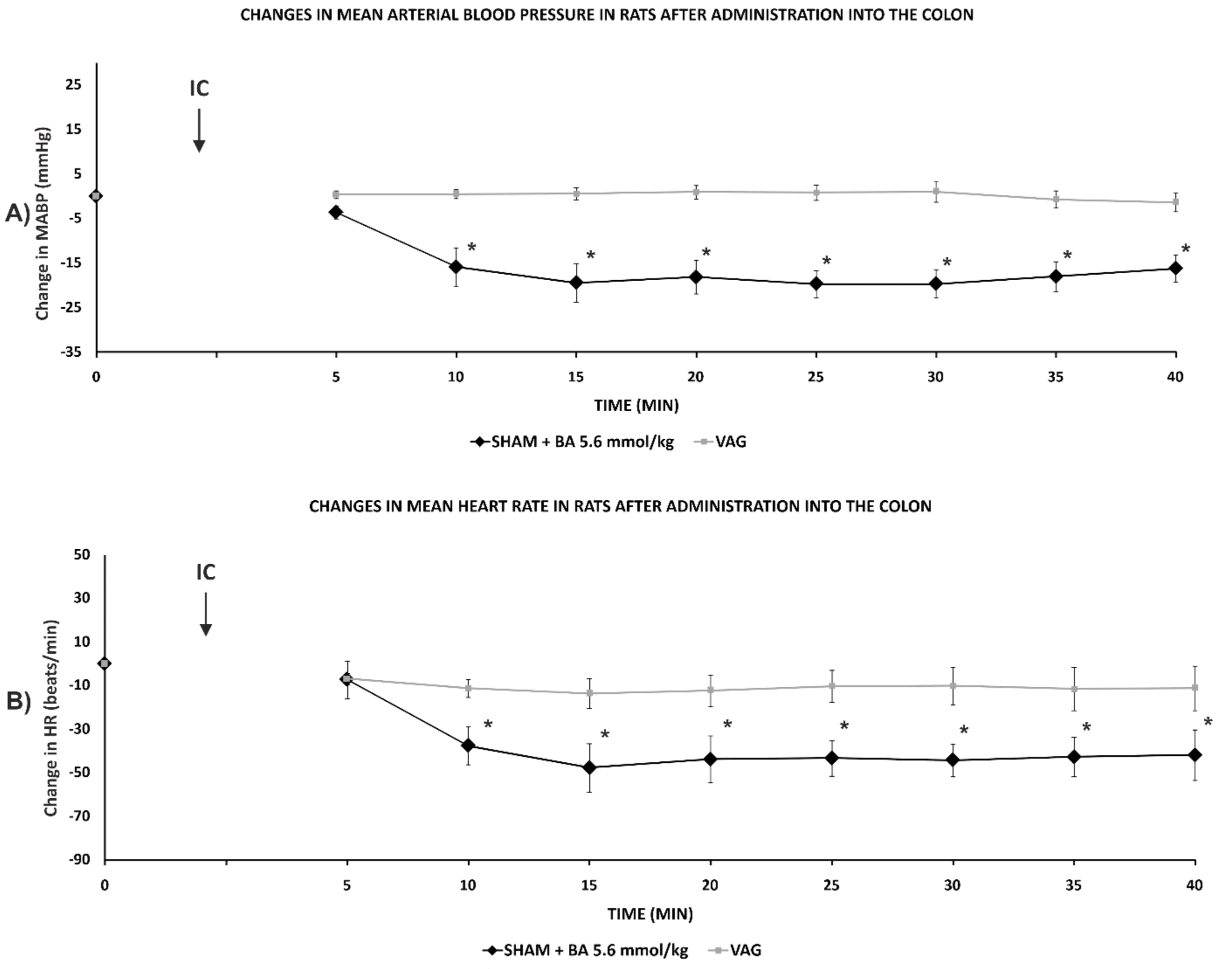

Figure S12.

[A] Changes in mean arterial blood pressure (ΔMABP, mmHg) and **[B]** heart rate (ΔHR, beats/min) in Wistar rats after administration into the colon (IC) of BA at a dose of 5.6 mmol/kg b.w after shame surgery. Means ± SE are presented.

* - vs Baseline

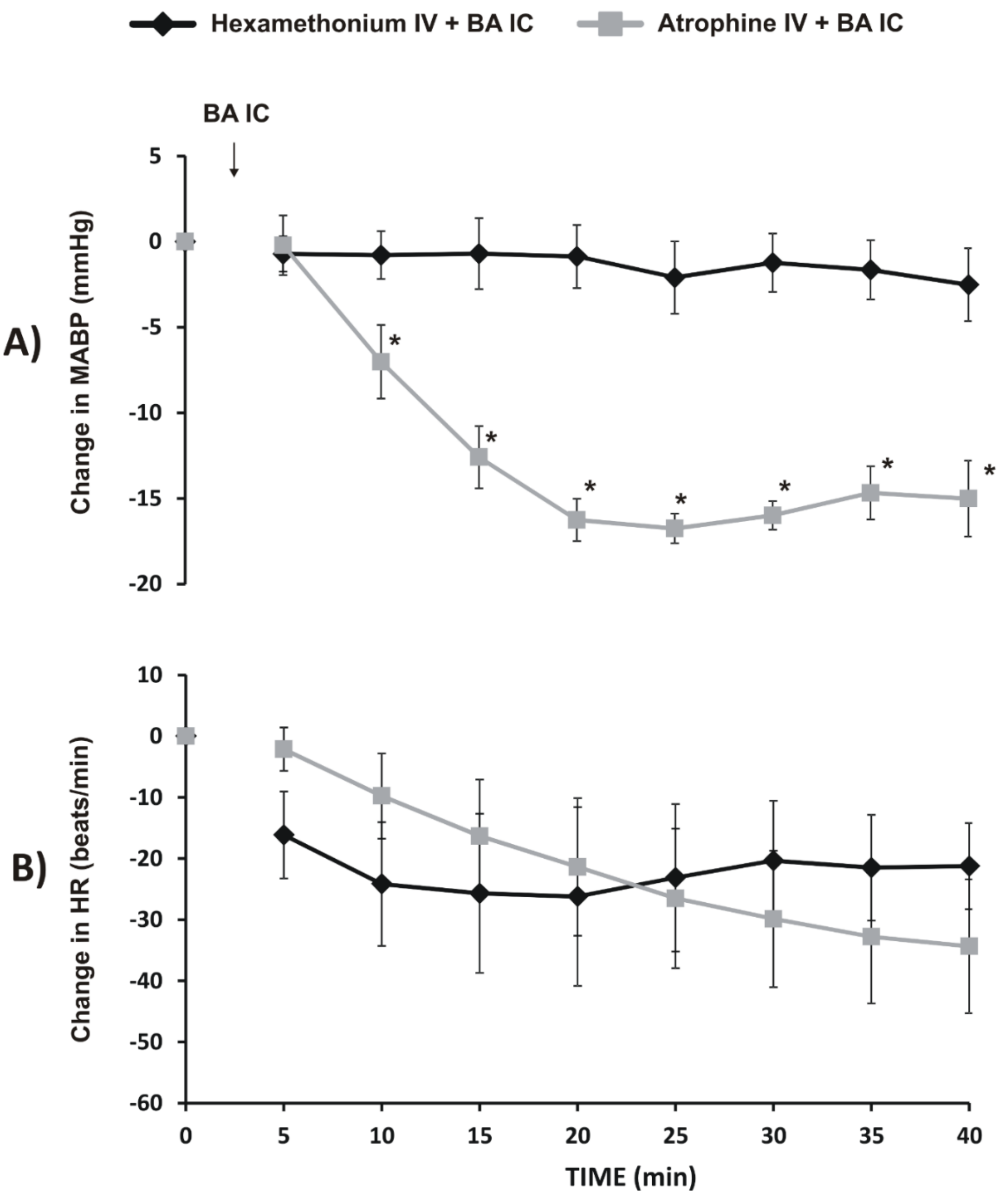

Figure S13.

Changes in mean arterial blood pressure (ΔMABP, mmHg) and heart rate (ΔHR, beats/min) in Wistar rats after the intracolonic administration of BA (BA IC) at a dose of 5.6 mmol/kg in rats pretreated with **[A]** hexamethonium (hexamethonium IV + BA IC) or **[B]** atropine (atropine IV + BA IC). Means ± SE are presented

*-p<0.05 - vs baseline.

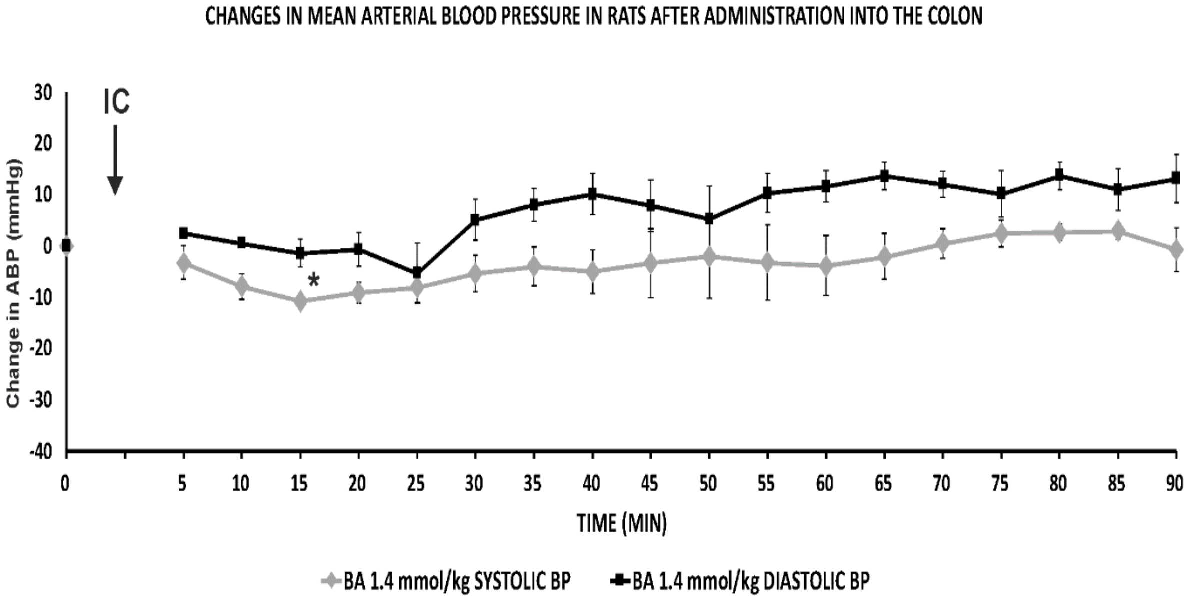

Figure S14.

Changes in arterial blood pressure (ΔABP, mmHg) in Wistar rats after the intracolonic administration (IV) of BA at a dose of 1.4 mmol/kg. Means ± SE are presented.

* - vs Baseline

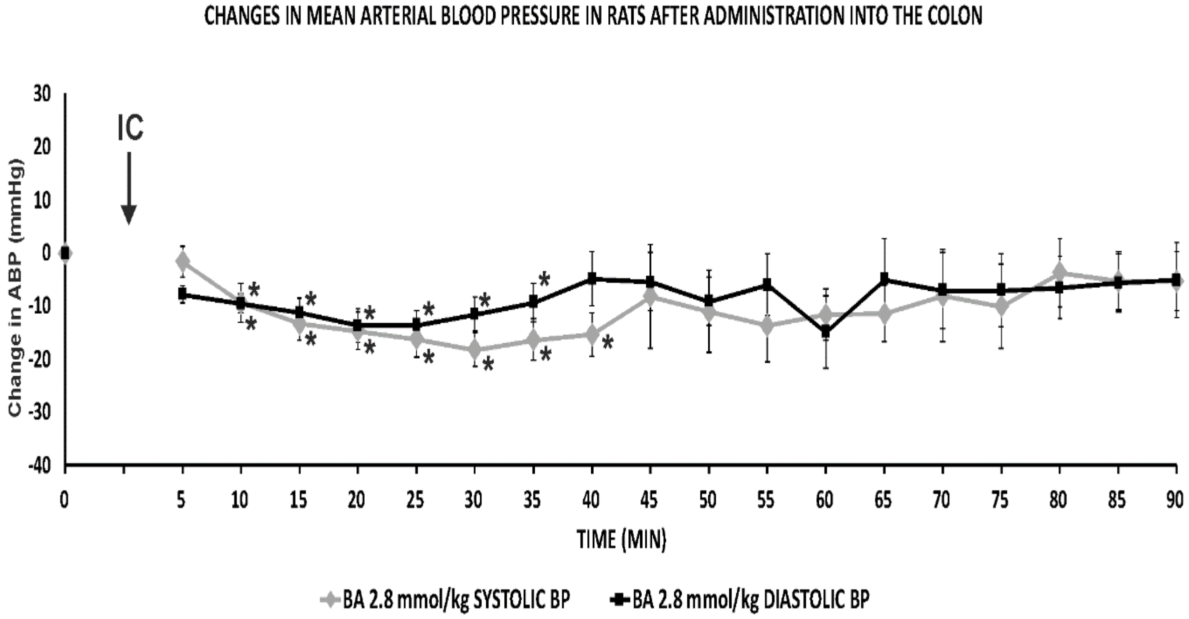

Figure S15.

Changes in arterial blood pressure (ΔABP, mmHg) in Wistar rats after the intracolonic administration (IV) of BA at a dose of 2.8 mmol/kg. Means ± SE are presented.

* - vs Baseline

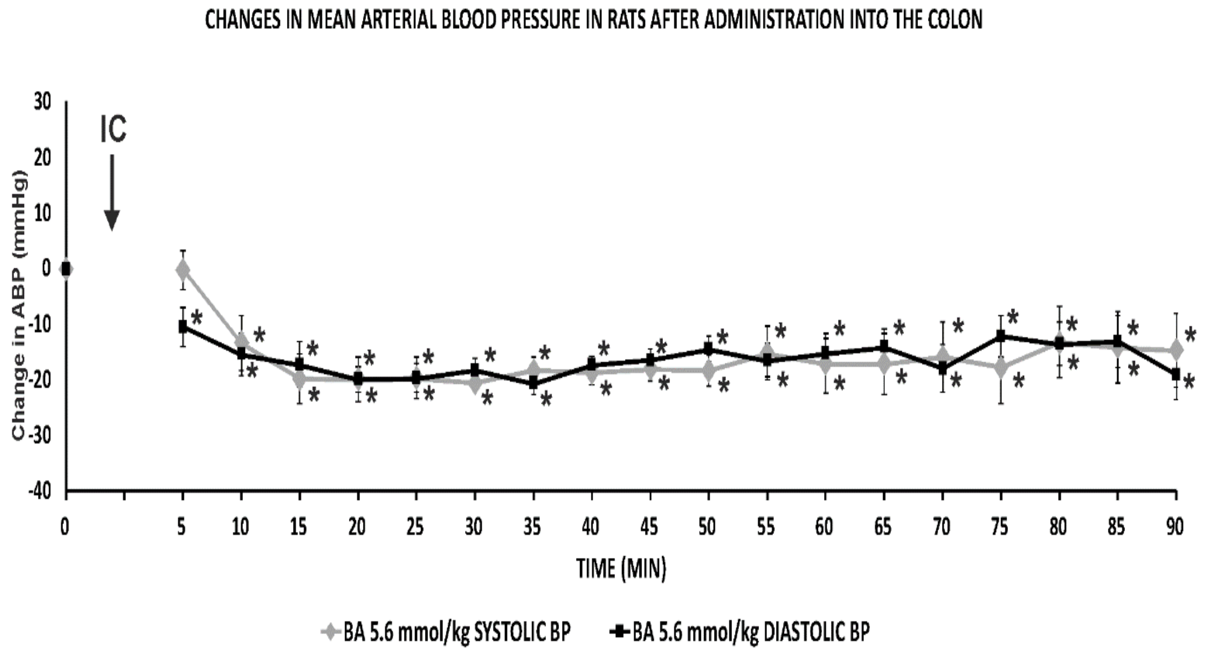

Figure S16.

Changes in arterial blood pressure (ΔABP, mmHg) in Wistar rats after the intracolonic administration (IV) of BA at a dose of 5.6 mmol/kg. Means ± SE are presented.

* - vs Baseline
